# Extraction of *Nannochloropsis* Fatty Acids Using Different Green Technologies: The Current Path

**DOI:** 10.3390/md21060365

**Published:** 2023-06-19

**Authors:** Sérgio Cruz Sousa, Ana Cristina Freitas, Ana Maria Gomes, Ana P. Carvalho

**Affiliations:** 1 CBQF—Centro de Biotecnologia e Química Fina, Laboratório Associado, Escola Superior de Biotecnologia, Universidade Católica Portuguesa, Rua Diogo Botelho 1327, 4169-005 Porto, Portugalapcarvalho@ucp.pt (A.P.C.); 2 REQUIMTE/LAQV—Instituto Superior de Engenharia, Instituto Politécnico do Porto, Rua Dr. António Bernardino de Almeida, 431, 4200-072 Porto, Portugal

**Keywords:** green extraction, disruption, polar lipids, polyunsaturated fatty acids, eicosapentaenoic acid

## Abstract

*Nannochloropsis* is a genus of microalgae widely recognized as potential sources of distinct lipids, particularly polyunsaturated fatty acids (PUFA). These may be obtained through extraction, which has conventionally been performed using hazardous organic solvents. To substitute such solvents with “greener” alternatives, several technologies have been studied to increase their extraction potential. Distinct technologies utilize different principles to achieve such objective; while some aim at disrupting the cell walls of the microalgae, others target the extraction per se. While some methods have been utilized independently, several technologies have also been combined, which has proven to be an effective strategy. The current review focuses on the technologies explored in the last five years to extract or increase extraction yields of fatty acids from *Nannochloropsis* microalgae. Depending on the extraction efficacy of the different technologies, distinct types of lipids and/or fatty acids are obtained accordingly. Moreover, the extraction efficiency may vary depending on the *Nannochloropsis* species. Hence, a case-by-case assessment must be conducted in order to ascertain the most suited technology, or tailor a specific one, to be applied to recover a particular fatty acid (or fatty acid class), namely PUFA, including eicosapentaenoic acid.

## 1. Introduction

Microalgae have long been known to be a source of several compounds with quite interesting properties. As such, microalgae or its extracts have diverse applications in different areas, such as human nutrition, feed in aquaculture, biofertilizers, treatment of effluents, and even in human health [1,2,3]. Bioactive compounds present in microalgae include lipids, namely polyunsaturated fatty acids (PUFA), which are probably the most studied amongst compounds, sterols, pigments, proteins, enzymes, vitamins and other compounds with biological activity [2,3,4].

*Nannochloropsis* is a genus of microalgae comprising seven described species, wherein individuals are characterized by being non-motile, presenting a spherical morphology and diameters ranging from 2 to 8 µm [2,5,6]. Furthermore, these microalgae are also widely recognized for presenting high PUFA contents, particularly that of the omega-3 fatty acid eicosapentaenoic acid (EPA, C20:5n3) [7,8,9,10,11,12]. As all microalgae, *Nannochloropsis* have different composition lipids, which may be divided into polar (phospholipids, glycolipids, and betaine lipids) and non-polar (acylglycerols, sterols and free fatty acids) [13,14]. The PUFA present in these microalgae are mainly located in polar lipids, namely phospholipids, while their content in neutral triacylglycerols (TAG) is lower [8,15]. This is an advantage, since omega-3 PUFA are more stable and possess higher bioavailability when in the form of polar lipids (particularly phospholipids) [12,16].

In order to obtain the compounds of interest from microalgae cells, present in the cell wall itself or intracellularly, extraction must be performed [3,17]. The main objective of extraction techniques is to obtain a high yield of the desired compounds without jeopardizing quality and activity, as well as to preserve co-products, minimize the amount of energy spent and waste generation, optimize the process (operational temperature, pressure, carrying capacity, side reactions and separations) and be scalable [18].

Traditionally, fatty acids were obtained from microalgae via the conventional solvent extraction techniques. These techniques include solid–liquid and liquid–liquid extractions, in which organic solvents, such as hexane, toluene, dichloromethane, acetone and others are utilized [18,19]. However, nowadays, there is a generalized opinion that the solvents used in the extraction processes should be safe, inexpensive and nontoxic [18,20,21]. Hence, recent studies have focused on the development of extraction technologies to obtain microalgae extracts without utilizing (or minimizing the use of) toxic organic solvents, or by substitution by “greener” solvents, such as ethanol and deep eutectic solvents [3,18,22,23]. Nonetheless, non-aggressive extraction conditions must be utilized, to not have a detrimental impact on the compounds present in the microalgae cells, such as the degradation of lipids, which may occur at high temperatures [24,25,26].

The extraction of biologically active compounds (namely fatty acids) from microalgae, such as *Nannochloropsis*, has been performed utilizing several different technologies which include ultrasounds [8,27], microwaves [28,29], super- and subcritical fluids [30,31], and high pressure [32], among others.

Extraction processes may entail a very important step, that is, the pretreatment of the biomass, to increase/improve the extraction yield, obtained by disrupting the cell wall [18]. Cell wall rigidity can inhibit conventional organic solvents, such as hexane, to enter the cell, preventing or hindering the contact between the solvent and the intracellular compounds [24]. As such, pretreatment makes the bioactive compounds present in the cells more bioavailable [26]. Cell wall disruption techniques can be of mechanical, chemical, physical or enzymatic nature, such as high pressure homogenization, alkaline lysis, ultrasonication, and enzymatic hydrolysis [33,34,35,36]. *Nannochloropsis* microalgae have been described as possessing a rigid, robust, cell wall [7,32], resulting from its composition, consisting of an outer hydrophobic trilaminar sheat algaenan-based layer and an inner cellulose-based layer (linked by struts to the plasma membrane), which hinder the extraction of intracellular compounds [2,7,20,26,35]. Thus, pretreatments to disrupt cell wall are desired/required to increase extraction yields.

**Figure 1:**
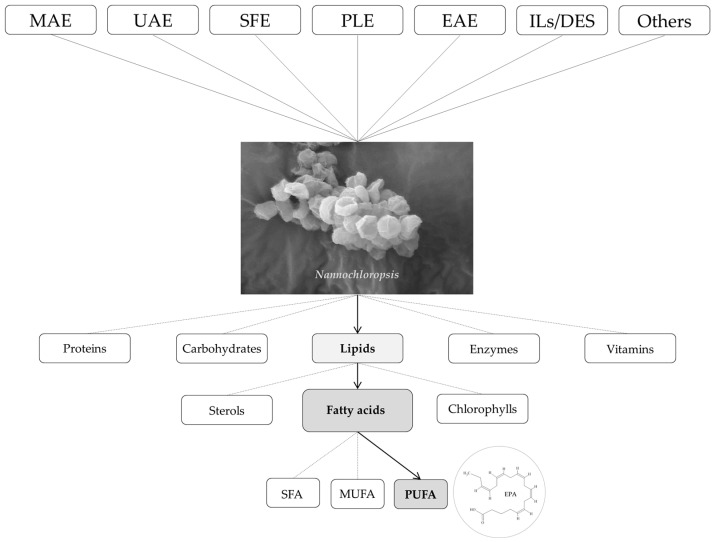
Technologies recently used to extract fatty acids from *Nannochloropsis*.

The current review provides an overview of the main extraction technologies utilized in the last five years to extract fatty acids from *Nannochloropsis* microalgae (Figure 1), as well as the principles/mechanisms responsible for the extraction effectiveness of each of those technologies.

## 2. Extraction Technologies

### 2.1. Microwave-Assisted Extraction (MAE)

Microwave technology can, as previously mentioned, be used to enhance yields of bioactive compounds by disrupting cell walls and is used as a complement in the extraction methodology.

Microwaves are the alternating current signals with frequencies varying from 0.3 to 300 GHz, which transform electromagnetic energy into heat with the polarity of compounds. Polar compounds will realign in the direction of the electric field and, when the microwave field alters, they will rotate in high speed. If ions are present, ions will migrate as the electric field alters. Electromagnetic energy is then transformed into heat by the friction between the compounds or the ions [37]. Microwave heating is a non-contact heat source, heating the overall target reactants simultaneously as compared to conductive heating [38]. The heat source can penetrate into the biomaterial, interacting with polar molecules, such as water, and heat the entire sample uniformly [39]. The increase in temperature will cause the evaporation of water molecules, which will apply pressure on the cell walls. This will rupture the cell walls, which will release the intracellular components into the medium. The utilization of microwaves also facilitates extraction as hydrogen bounds are disrupted and dissolved ions, by migration, increase the penetration of solvent into the matrix [18]. Microwave-assisted extraction is considered to be a rapid and cost-effective method of obtaining bioactive compounds [18,37,39], and has been frequently used to extract different compounds from microalgae.

Regarding the extraction of fatty acids from *Nannochloropsis*, microwaves were usually applied at a frequency of 2.5 GHz, potencies below 800 W, temperatures lower than 100 °C and during a maximum of 30 min. One example, in which the highest potency was utilized, is the study of Quesada-Salas et al. (2021) [29], which compared MAE with UAE (ultrasound-assisted extraction) or bead milling to extract lipids from two *Nannochloropsis* species, namely *N*. *gaditana* and *N*. *oceanica*. The authors studied the temperature (between 50 and 100 °C) and time (between 5 and 25 min) of treatment, finding differences in the impact according to the species. By using quadratic models to predict the lipid extraction yields, the authors found that although all parameters impacted the lipid yields from both species, some parameters impacted them differently. Regarding temperature, it positively correlated with *N*. *gaditana* lipid extraction, while showing no interaction with time. In *N*. *oceanica*, temperature presented a positive effect on lipid yield only to a certain threshold, after which it would present a negative effect, possibly due to lipid degradation at higher temperatures. Concerning the interaction with time, it was found that in *N*. *oceanica*, they do interact, however, the behavior was distinct depending on the specific temperature. The study also showed that distinct disruption technologies may change the fatty acid profile (namely that of EPA) of the extract of a specific species, and that this effect is species-dependent, since it was only observed regarding *N*. *oceanica*. Concerning the differences between technologies, MAE presented the highest yield (including EPA) from *N*. *oceanica*, while UAE was the most efficient for lipid extraction from *N*. *gaditana*, presenting lipid recoveries of 49 and 21.7% dry weight (dw), respectively.

Another study regarding *Nannochloropsis* in which MAE was utilized, in this case at low potencies (≤100 W), was that of Zghaibi et al. (2020) [40]. In that research work, microwaves were associated with brine (NaCl), and temperature (between 60 and 100 °C) and extraction time (between 1 and 30 min) were evaluated for the impact on lipid extraction from *Nannochloropsis* sp. Results showed the highest PUFA percentage (44.1% dw, corresponding to 25.2 mg g^−1^) was when temperature was the highest, which authors speculated could have resulted from a larger extension of ruptured cell walls (in which high amounts of PUFA are present). Regarding time, the authors found a similar result, with longer extraction times presenting higher amounts of those fatty acids, which was also observed for omega-3 fatty acids and monounsaturated fatty acids (MUFA). Those results were corroborated via scanning electron microscopy, which revealed larger cell damages in cells exposed to higher temperature and/or longer extraction time.

Table 1 presents other studies performed within the last five years in which microwaves have been applied either as a single treatment testing different potencies, extraction times (up to 30 min) and temperatures (up to 100 °C), or in combination with other technologies, namely ultrasounds [41] or ionic liquids [42], to obtain lipids from *Nannochloropsis* microalgae.

### 2.2. Ultrasound-Assisted Extraction (UAE)

Ultrasound-assisted extraction is another technology utilized to disrupt cell walls, and consequently increase extraction yields, which is sometimes used simultaneously with MAE.

This technology is based on the cavitation phenomenon. When a liquid is submitted to ultrasounds, cavitation bubbles are generated, which can create implosive collapse [46]. The intense sonication of liquid generates soundwaves that propagate into the liquid, resulting in alternate high-pressure and low-pressure cycles. Cavitation is the phenomenon resulting from the violent collapse of small vacuum bubbles, generated in the low-pressure cycle, during the high-pressure cycle. During cavitation, shearing forces are formed around the cells by the high pressure and high speed liquid jets, resulting in the cell structure being “mechanically” broken, thereby improving material transfer [39]. This reduces particle size and increases the contact between the solvent and the compounds to be extracted [18]. The enhancement in extraction yields is attributed to the microstreaming and heightened mass transfer by cavitation and bubbles collapse, which result in the destruction of the cells [47]. This technology can, as previously mentioned, improve extraction yields and reduce the amount of solvent utilized, and the extraction time and costs, as there is a reduction in the temperature needed for the extraction process [47,48].

Concerning *Nannochloropsis*, ultrasounds were commonly utilized at frequencies of 20 or 37 kHz, and potencies between 100 and 140 W. Regarding other parameters, amplitude, temperature and time were extremely variable. Similar to MAE, the latest research works have explored ultrasounds’ association with other technologies and solvents (Table 2). One such example is the study of Guo et al. (2022) [27], in which ultrasounds were associated with a switchable hydrophilicity solvent (N, N, N′, N′-tetraethyl-1,3-propanediamine) to rupture the cell wall of *N*. *oceanica*. Exposure of the microalga to the solvent per se was sufficient to damage the cell wall, by reducing its thickness from 141 to 68.6 nm, and to originate lipid leakage. Nonetheless, applying ultrasounds further increased the extent of the damage, and consequently of the extraction yield. Indeed, ultrasonication of the solvent/biomass mixture presented a synergistic effect, which lead to a disruption of the cell wall, allowing for a high lipid extraction efficiency (98.2%). However, a differential fatty acids profile was obtained, particularly in PUFA, where in comparison with the conventional Bligh and Dyer (1959) [49] method (which utilizes a solvent mixture of chloroform/methanol, 1:2, *v*/*v*), the combined extraction process decreased the percentage of C18:2 and C18:3 in the extract, although EPA was increased.

In turn, Blanco-Llamero et al. (2021) [50] combined ultrasounds with commercial enzymes (namely a mixture of carbohydrases (Viscozyme^®^), cellulase (Celluclast^®^), and protease (Alcalase^®^)) to disrupt the cell wall of *N*. *gaditana*, to extract lipids. The authors found that ultrasounds were able to increase the extraction efficiency of the enzymes, and that an ultrasound-assisted enzymatic treatment (with the combination of all three enzymes), in which enzymatic activity was performed in an ultrasound bath, was able to yield the highest amount of lipids (28.9% dw), doubling the amount obtained with non-pretreated biomass (~14.5% dw). That treatment was also the one in which the extract presented the highest amount of polar lipids (45.66% glycolipids and 2.51% phosphatidylethanolamine), in comparison with the non-pretreated biomass (35.42% glycolipids and 1.79% phosphatidylethanolamine).

Ultrasounds were also studied by Figueiredo et al. (2019) [8] to increase the extraction efficiency of ethanol, with the aim of obtaining EPA-enriched lipid extracts from *N*. *oceanica*. The study showed that, by utilizing ultrasounds, the amount of EPA extracted using ethanol could be increased. Moreover, comparison between applying ultrasounds with a bath or with a probe showed that the latter was more efficient, allowing for the amount of PUFA and EPA extracted to be increased by 47 and 35%, respectively, when compared to extraction solely with ethanol.

This technology has also been associated with others, such as low-temperature hydrothermal liquefaction [51] or microwaves [41], as presented in Table 2.

**Table 2:** Extraction of lipids and fatty acids from *Nannochloropsis* using ultrasound-assisted extraction reported within the last five years.

US/other	Species	Solvent	Operational Conditions			Yield						References
						Lipid	Fatty Acids					
			Used	Tested	Optimum		SFA	MUFA	PUFA	Omega-3	EPA	
US	*N*. *gaditana*	CHCl_3_:MeOH (2:1)	130 W; 20 kHz	50–80% amplitude; 10–30 min	80% amplitude; 30 min	21.7% (dw)	―	―	―	―	―	[29]
	*N*. *oceanica*					45.4% (dw)	―	―	―	―	―	
US + Switchable hydrophilicity solvent	*N. oceanica*	TEPDA	0.5 W/mL, 20 kHz, room temperature	30–180 min	120 min	98.2% (wet weight)	―	―	―	―	―	[27]
US + Celluclast + Viscozyme	*N. gaditana*	―	140 W; 37 kHz	2–6 h (ultrasounds)/pH 4–8; 35–55 °C (enzymatic pretreatment)	55 °C, pH 5.0, 6 h	24.2%	―	―	―	―	―	[50]
US + Celluclast + Viscozyme + Alcalase						28.9%	―	―	―	―	―	
US	*N. oceanica*	EtOH	130 W; 20 kHz; 70% amplitude; 8 min	Ultrasound probe; ultrasound bath	Ultrasound probe	60.3% (extract dw)	27.4 mg g^−1^ (dw)	24.6 mg g^−1^ (dw)	21.8 mg g^−1^ (dw)	―	15.4 mg g^−1^ (dw)	[8]
US + Low-temperature hydrothermal liquefaction	*Nannochloropsis* sp.	Dichloromethane	100 W; 60 min	30–90 s (sonication); 210–250 °C (HTL)	90-s sonication time, 250 °C	28.9% (dw)	―	―	―	―	―	[51]
―	*N. gaditana*	2-MeTHF:EtOH (1:3)	37 kHz; 50 °C; 30 min	EtOH; 2-MeTHF; hexane:EtOH (3:4); 2-MeTHF:EtOH (1:1, 1:2, 1:3, and 1:4)	2-MeTHF:ethanol (1:3)	16.3% (dw)	―	―	―	―	―	[23]
US + MW	*Nannochloropsis* sp.	MeOH	―	MeOH (10–30 mL); Ultrasounds and Microwaves Power (100–140 W); reaction time (3–7 min)	30 mL MeOH, 140 W (microwaves), 140 W (ultrasounds), 7 min	22.8% (dw)	66.5% (TFA)	20.7 (TFA)	12.7% (TFA)	―	―	[41]

US—ultrasounds; MW—microwaves; SFA—saturated fatty acids; MUFA—monounsaturated fatty acids; PUFA—polyunsaturated fatty acids; EPA—eicosapentaenoic acid; TFA—total fatty acids; EtOH—ethanol; MeOH—methanol; 2-MeTHF—2-Methyltetrahydrofuran; TEPDA—N, N, N′, N′-tetraethyl-1,3-propanediamine; CHCl_3_—chloroform; dw—dry weight.

### 2.3. Supercritical Fluid Extraction (SFE)

Supercritical fluid extraction (SFE), together with pressurized liquid extraction (PLE), is probably the most widely employed extraction technique for obtaining bioactive components from natural sources [52]. Supercritical fluid extraction utilizes the solvents above their critical pressures and temperatures [18,52]. As the solvent power of a supercritical fluid is a function of density, it can be varied by changing the extraction pressure and temperature, enabling it to be suitable as an extraction solvent [39]. In the conditions utilized in SFE, supercritical fluids (SCFs) possess particular physicochemical properties between gases and liquids, generally acquiring higher density than a gas, yet maintaining similar viscosities and diffusivities [52]. The density of the SCF is like that of a liquid, and it can be altered by changing the temperature and pressure. The low viscosity and high diffusivity of SCFs generates better transport properties, when compared to liquids [18]. Although different solvents can be utilized, the most utilized solvent is carbon dioxide (CO_2_), due to its moderate critical pressure (7.4 MPa) and low critical temperature (31.1 °C) [39]. Supercritical CO_2_ (SC-CO_2_) has several advantages, as it has mild critical conditions, and is nontoxic, nonflammable, nonexplosive and noncorrosive. Additionally, it is easily available and cheap, and is easily separated from the extract, inert to the product. Carbon dioxide, being a gas at room temperature, can be easily removed from the extract, when compared to other extraction techniques [18,52]. Another advantage is that the properties of SCFs can be adjusted with pressure and temperature changes, which directly influences density, making the technique very selective, which is a major advantage when the objective is the extraction of compounds from complex matrices. This technique has also the advantage of the possibility of, during decompression, performing fractioning just by utilizing two or more decompression steps, which is useful to separate components in the extract [52].

In addition to the abovementioned advantages, like every technique, SFE also has disadvantages. The main disadvantage of SC-CO_2_ is its low polarity. This property limits the compounds that can be extracted using this technique, which alone may not be able to extract polar compounds. However, this issue can be overcome by the addition of cosolvents (modifiers), which are employed at small proportions (1–10%), during the extraction process [52]. The cosolvents are solvents with higher polarity, which changes the polarity of the SCF and thus increasing the solvating power, thereby increasing the range of compounds that can be extracted [18,52]. This was the case in Askari et al.’s (2022) [30] study, in which SC-CO_2,_ in combination with *n*-hexane, was utilized as cosolvent to extract lipids from *N*. *oculata*. The authors assessed the impact of the cosolvent’s presence, as well as that of temperature (between 35 and 75 °C) and pressure (between 150 and 550 bar), on the lipid and PUFA yields, finding that lipid yield and extraction kinetics were directly correlated with both temperature and pressure, independent of the other condition. This means that lipid yield and extraction kinetics increased with temperature at low or high pressure, and a similar behavior was observed regarding pressure in relation to temperature. The study also revealed that the presence of the cosolvent positively impacted both the amount of lipids extracted, as well as the rate at which they were obtained. Furthermore, lacing SC-CO_2_ with *n*-hexane also resulted in a distinct fatty acid profile of the extract, doubling the amount of EPA, and increasing total PUFA content to nearly double of the saturated fatty acids (SFA), which was previously 1.5-fold higher than PUFA.

Leone et al. (2019) [53] explored the use of SC-CO_2_ to extract omega-3 fatty acids from *Nannochloropsis* sp., pre-treated mechanically with diatomaceous earth. Results showed that, according to the pressure and temperature of the process, extraction selectivity could be tailored towards obtaining more EPA or docosahexaenoic acid (DHA). Specifically, when utilizing the highest pressures (550 bar) and temperatures (75 °C), EPA yield was increased, while the highest DHA recoveries were obtained at milder conditions (400 bar and 50 °C). The impact of the CO_2_ flow rate on both EPA and DHA yields was also assessed, and the authors determined that they were directly correlated, since an increase in both omega-3 fatty acids yields (increase of ~45 and 70%, respectively) was observed when the flow rate was doubled.

Further examples of SFE (utilized at pressures in the range of 100–550 bar and temperatures varying from 40 up to 150 °C), alone or in combination with other strategies, applied to *Nannochloropsis* microalgae for lipids extraction, focusing on fatty acids, are presented in Table 3.

### 2.4. Pressurized Liquid Extraction (PLE)

Pressurized liquid extraction (PLE), also referred to as pressurized fluid extraction (PFE), pressurized hot-solvent extraction (PHSE) or accelerated solvent extraction (ASE), is an extraction technology based on the utilization of pressurized solvents at high temperatures, although always below their critical points, under conditions that maintain the solvents in the liquid state during the extraction process. When water is utilized as the extraction solvent, the general principles and instrumental requirements are the same, although other important parameters have significant influence, and the technology can be denominated as subcritical water extraction (SCWE), superheated water extraction (SHWE) or pressurized hot-water extraction (PWE) [52].

Pressurized liquid extraction conditions provide an enhanced mass-transfer rate, an increased solubility of the compounds to be extracted, and a decrease in solvent viscosity and surface tension [23,52]. The lower solvent viscosity and surface tension will allow the solvent to penetrate more easily into the matrix, reaching deeper areas and increasing the surface contact, which will improve the mass transfer to the solvent, resulting in an increased extraction rate [52]. As previously mentioned, when water is utilized as the solvent, the extraction is also affected by the dielectric constant (ε) of water. When water is heated at high temperatures while remaining in the liquid state, ε, which is a measure of the polarity of the solvent, is significantly reduced [31,52]. If this value is decreased to values close to the ones of organic solvents (when heated), water can be presented as a useful alternative. Even though this may not be possible for all applications, SWE can be seen as the “greenest” of the PLEs [52].

When compared to the conventional extraction processes, PLE presents numerous advantages, such as higher selectivity, shorter extraction times, faster extraction processes and smaller amounts of organic solvents [18,52]. Additionally, the possibility of automation is a further advantage of this technique, since it helps to reduce variations between extractions, which increases reproducibility [52].

Pertaining to pressurized liquids for extraction of fatty acids from *Nannochloropsis*, Blanco-Llamero and Señoráns (2021) [23] utilized the technology in combination with bio-based solvents to achieve less environmentally detrimental alternatives to extract omega-3 fatty acids from *N*. *gaditana*. The authors tested 2-methyltetrahydrofuran (2-MeTHF) as the extraction solvent, as well as its combination with ethanol, in a 1:3 (2-MeTHF:ethanol) ratio. Results showed that the combination of solvents yielded the highest lipid amount (16.32% dw) and that PLE with ethanol per se originated the extract with the highest glycolipids content (42.99% of extract), which the authors attributed to the higher polarity of the solvent. Nonetheless, the fatty acids profiles revealed that, concerning PUFA and EPA percentages (~48 and 36% of total fatty acids (TFA), respectively), no significant differences were obtained when the extract was obtained using ethanol as the only solvent, or when in combination with 2-MeTHF.

Regarding the specific case of water, Eikani et al. (2019b) [58] found that, when extracting lipids from *N*. *salina*, in comparison with the conventional Folch et al. (1957) method [59] (with corresponding solvent mixture of chloroform/methanol, 2:1, *v/v*), the extract possessed lower PUFA amount (11.41 and 19.98% of TFA, respectively). The authors ascribed the difference to a thermal degradation that may have had occurred due to the high temperatures utilized in SCWE, and to a possible thermal oxidation, which could have converted those PUFA to lower double bond PUFA, or even to saturated ones. In a distinct study [31], the same research group reported that utilizing ethanol as a solvent modifier in SCWE yielded an extract with higher lipid and PUFA contents. The authors also found that presence of the cosolvent allowed the extraction process to be performed at lower temperatures (90 °C), which may have decreased the extension of the abovementioned phenomena, or even prevent them from occurring.

Further examples of the technology applied recently to *Nannochloropsis* can be found in Table 4.

### 2.5. Enzyme-Assisted Extraction (EAE)

Enzyme-assisted extraction (EAE) is yet another technology/technique which has been utilized to obtain fatty acids from microalgae. As previously mentioned, microalgae possess cell walls which, dependent on their composition, may hinder the access of the extraction solvent to the intracellular compounds. In this sense, there is the need to rupture or, at least, disrupt the cell wall so that extraction of such compounds may be achieved. The algaenan/cellulose wall of *Nannochloropsis* is particularly resistant to chemical or mechanical treatments [60] and in many cases there is a need to apply combinations of these to increase the extraction potential as described in the previous sections. A rather promising alternative strategy to overcome this constraint is the use of enzymes which, according to their nature, may hydrolyze the cell wall structural components. This will damage the cell wall integrity, thereby providing easier access of the extraction solvent to the intracellular compounds, as well as promoting their leakage [61,62]. In this sense, for *Nannochloropsis* microalgae, a cellulase can be applied, envisioning the degradation of the inner cellulose-based layer. The main treatment parameters which impact the enzyme, and consequently extraction efficiency, are the enzyme dosage, pH, temperature, time, and the homogenization (agitation) speed [20,61]. The combination of distinct enzymes, which is a strategy utilized to increase extraction yields [20], must be carefully evaluated, since their interaction may have an antagonistic effect, opposite to the desired synergistic one [61].

Recently, Zhao et al. (2022) [20] tested the activity of several enzymes, namely cellulase, laccase, pectinase, mannanase and xylanase on *N*. *oceanica*, as a pretreatment for ethanol extraction of lipids. After determining the two enzymes that yielded the highest lipid amounts (cellulase and laccase), whose extracts also presented the highest EPA contents, the authors combined the two enzymes. Optimization of enzymatic treatment was performed for enzyme dosage, enzymes ratio, buffer pH, temperature and time. The extract obtained through the combination of the two enzymes in a cellulase:lacase (2.5:1) ratio presented the highest EPA content, which increased 1.5-fold, compared with that obtained from untreated cells. Regarding the lipid classes, betaine lipids and free fatty acids were the ones positively impacted (greatly increased) by the enzymes pretreatment, while neutral lipids, phospholipids and glycolipids’ contents decreased.

Qiu et al. (2019) [63] also assessed the impact of four enzymes, namely cellulase, hemicellulase, papain and pectinase, on lipid extraction from wet *Nannochloropsis* biomass, in an enzyme-assisted three phase partitioning, in which *tert*-butanol was utilized as the extraction solvent. The authors assessed the impact of several parameters, such as enzyme type and slurry/*tert*-butanol ratio, among others. In accordance with the findings of the abovementioned study of Zhao et al. (2022) [20], and with the known cellulose-based inner layer of the cell wall, the enzyme that presented the best result was cellulase. Extraction using the cellulase-assisted three phase partitioning yielded an extract with increased PUFA and EPA contents (19.91 and 13.98% of TFA, respectively), in comparison with those obtained without enzymatic hydrolysis (16.23 and 12.95% of TFA, respectively). Moreover, under the optimized conditions (20% ammonium sulphate, 6–7 pH, 1:2 slurry/*tert*-butanol ratio, 70 °C for 2 h), the cellulase-assisted three phase partitioning extraction system was able to extract 90% of total fatty acids after two extraction cycles, and it was demonstrated that it was scalable, as it presented similar results in a laboratory scale of 20 L. In this sense, the enzyme-assisted three phase partitioning was revealed to be a promising strategy to obtain fatty acids from wet *Nannochloropsis*.

Further studies pertaining to EAE, with more in-depth explanation of the parameters and associated ranges assessed therein, are presented in Table 5.

### 2.6. Ionic Liquids (ILs) and Deep Eutectic Solvents (DES)

Recently, there has been a demand for solvents able to extract lipids, among other compounds, from distinct “matrices”, including microalgae, without having such a detrimental environmental impact as the conventionally utilized organic solvents. This has prompted researchers to explore other types of solvents, which include ionic liquids (ILs) and deep eutectic solvents (DES).

Ionic liquids are a class of solvents which, as aforementioned, have been studied as alternatives to the conventionally used solvents to extract several compounds from microalgae. The ILs are solutions of salts that present melting temperatures below 100 °C, some of which may still even be liquid (molten) at room temperature, and their composition comprises both anions and cations, hence their designation [7,65,66,67]. These solvents’ properties can be manipulated by combination and permutation of the anions and cations comprised therein, which endow solvents with distinct polarity, thermal stability, hydrophobicity and viscosity, that can be tailored according to the specific goal for which they are intended [3,65,67]. Furthermore, within ILs there is a subclass denominated switchable solvents, of which there are two types, namely switchable polarity solvents (SPS) and switchable hydrophilicity solvents (SHS), which can reversibly change the characteristics in response to a stimulus/trigger [66,67,68].

Although ILs have been considered in “green” extractions, there are significant environmental concerns regarding the utilization of these solvents due to the inefficient biodegradation and the potential use and production of toxic reagents in the synthesis of some ILs [67,68]. Nevertheless, they have been studied with regard to lipid extraction from microalgae, as in Shankar et al. (2019) [69], in which protic ILs (a subtype of ILs) have been utilized to extract lipids from *N*. *oculata*. The authors found that, in comparison with the conventional Bligh and Dyer (1959) method [49], extraction via ILs (in combination with a posterior microwaves treatment), in particular butyrolactam hexanoate, increased lipid yield by 34.9%, with a lower content of pigments, which is a positive trait when fatty acid extraction is concerned. Moreover, the study revealed that extraction was more efficient when biomass was hydrated, which is also favorable to the implementation of the technology, since a drying step is circumvented.

As previously mentioned, DES (and natural DES, which are of a natural origin) are another type of solvent which have attracted the attention of researchers [3,70,71]. These comprise hydrogen-bond acceptors (HBA) (organic salts, such as choline chloride) and hydrogen-bond donors (HBD) (e.g., sugars or organic acids) [3,70]. Moreover, they present several properties which confer a “greener” status, in comparison with ILs and conventional organic solvents, which include biodegradability, low toxicity or lack thereof, easy synthesis and safety (nonvolatility and nonflammability). Despite the difficulty of separation from the extracted compounds, due to nonvolatility, since they are nontoxic, DES, and specifically natural DES, may be directly incorporated in food products, which also benefits the production as a purification step will not be required [70,71]. Similar to the abovementioned regarding ILs, switchable DES have also been developed to address cases in which separation of the compounds from the solvents are required [71].

Concerning *Nannochloropsis*, Cai et al. (2021) [71] studied the impact of DES on the extraction of lipids from *Nannochloropsis* sp., utilizing a three-phase partitioning system, and comparing the extraction with that performed using *t*-butanol. The authors found that the amount of lipids extracted using the DES, as well as the PUFA and EPA contents of such extracts, were higher (1.12 and 5.59-fold, respectively) than in the extract obtained using *t*-butanol. Moreover, DES was shown to retain considerable reusability, only losing 10% of lipid recovery after the fifth cycle was utilized.

Recent reports of lipid extraction from *Nannochloropsis* in which ILs or DES were studied are presented in Table 6.

**Figure 2:**
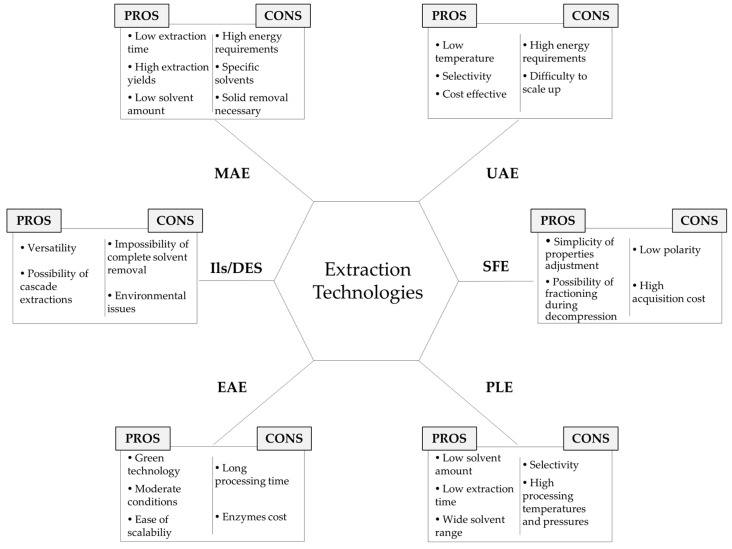
Pros and cons of the distinct extraction technologies.

An overview of the advantages and drawbacks of the different extraction technologies addressed so far is presented in Figure 2.

### 2.7. Others

In addition to the aforementioned technologies, a myriad of other solutions, some more innovative than others, have been studied to extract lipids, including fatty acids, from *Nannochloropsis* microalgae. Wang et al. (2018) [73] explored the effect of screw extrusion on *N*. *oceanica* cell integrity and lipid recovery, and found that the treatment increased the amount of fatty acids, including PUFA, subsequently extracted using hexane. Moreover, a balance between screw speed and feed moisture was shown to be critical to achieve the highest yields. Quesada-Salas et al. (2021) [29] also assessed, among other technologies, the impact of mechanical disruption by bead milling on *N*. *oceanica* and *N*. *gaditana* cells, followed by lipid extraction using a chloroform/methanol solvent mixture. The study revealed no effect of the flow rate variation (between high and low) on lipid yield, however, bead size was shown to significantly impact the extraction, with smaller beads being able to disrupt over two times the number of cells disrupted by the larger beads. Bead milling did not significantly change the fatty acid composition of the extracts of *N*. *gaditana*, although an increase in unsaturated fatty acids (UFA) and EPA was observed in the *N*. *oceanica* extracts.

Chemical methods have also been applied to enhance the extraction of fatty acids from *Nannochloropsis*. Potassium hydroxide (KOH) was utilized by Park et al. (2020) [74] to assist the solvent extraction of lipids from *N*. *oceanica*. The study showed that inclusion of KOH in the extraction process enabled the removal chlorophyll from the extract, which in turn resulted in an increased amount of fatty acid methyl esters (FAME). This resulted in an extract more suited for biodiesel production, and which could further be separated from the EPA comprised therein, so that it could be utilized in other products. One other chemical approach is osmotic shock. Halim et al. (2021) [75], aiming to extract lipids from *N*. *gaditana*, explored hypotonic osmotic shock as a method to induce cell rupture. Opposite to the expected/desired outcome, exposure of the marine microalga biomass to a fresh water hypotonic solution did not rupture the cells. Nonetheless, the authors found that there was indeed some damage to the cell walls, which enhanced the extent of the rupture resulting from the subsequent disruption techniques, in that specific case, high pressure homogenization, that allowed for a higher lipid yield.

Physical processes are likely the most studied regarding fatty acids extraction from *Nannochloropsis*. Lorente et al. (2018) [76] explored steam explosion as a pretreatment to diminish structural integrity of the cells, and consequently enhance lipid extraction from *N*. *gaditana*. The technology was able to disrupt the cell walls, and increase the amount of lipids extracted using hexane as extraction solvent by 8-fold, thereby resulting in a yield of ca. 80% as compared to the conventional Bligh and Dyer (1959) method [49]. Matos et al. (2019) [77] utilized non-thermal plasma to induce the rupture of *N*. *gaditana* cell walls, and found that the pretreatment to double the lipid yield, in comparison to untreated biomass, was similar to the obtained yield when microwaves were applied instead. The study also revealed that the fatty acids profile of the plasma-treated extract was distinct from the untreated, since the amount of omega-3 PUFA was decreased, while the SFA increased, which could be beneficial when biodiesel production is envisioned. Hydrodynamic cavitation is one other technology, which was utilized by Setyawan et al. (2018) [78] for lipid extraction from *Nannochloropsis* sp. The study showed that the yield was dependent on several parameters, namely the level and number of cavitation, microalga concentration, specific energy, and temperature. However, the authors did not determine the fatty acids profiles (which should be further explored), and therefore it is not possible to assess to what end such treatment may be indicated. In turn, hydrothermal disintegration was applied by Kröger et al. (2018) [26] to *N*. *oculata*, which obtained a 2-fold increase in lipid yield when compared with direct extraction, as well as an increase in C18:1 fatty acid while C18:0 and C18:3 contents were decreased (unusually, EPA contents were not reported). Teymouri et al. (2018) [79] studied flash hydrolysis to enhance the extraction of lipids from *Nannochloropsis* sp. and found that the treatment did increase the amount of lipids extracted. However, regarding lipid profile, it was determined that PUFA content decreased, with EPA being 4-fold higher in raw biomass which, therefore, resulted in a biodiesel-oriented extract. A shear-assisted process also was assessed by Kwak et al. (2020) [80] regarding lipid extraction from *Nannochloropsis* sp., and results showed that high lipid and EPA yields were achieved with minimal volume of extraction solvent. The technology was regarded as a viable alternative to several two-step extraction systems since it presented the lowest specific energy consumption. An extraction technology based on pulsed electric fields (PEF), namely high voltage electric discharge (HVDE), was utilized by Zhang et al. (2020) [6] to obtain bio-molecules from *N*. *oculata*, which included lipids. The authors found no significant differences between treated and untreated samples in regard to lipid yield, however, the highest EPA content was found in the extract from an untreated sample. In this sense, this technology was not indicated toward obtaining that specific omega-3 fatty acid. Nonetheless, HVDE treatment did increase the yields of palmitic (C16:0) and palmitoleic (C16:1n7) acids. Pulsed electric fields is a promising technology, worth of being more extensively explored to extract fatty acids from *Nannochloropsis*, since it has already been demonstrated to be efficient in increasing lipid extraction yields in other microalgae [81,82,83,84], and it was also utilized to obtain other compounds from microalgae of the *Nannochloropsis* genus [85,86]. Within physical processes which may be applied to extract *Nannochloropsis* fatty acids, high hydrostatic pressure has recently been reported to, independently or in combination with other technologies [32,87,88,89], enhance the extraction of PUFA, and specifically EPA [32,89], thus supporting the use of greener solvents than the conventional organic ones.

An altogether distinct approach was that of Halim et al. (2019) [90], which explored a mechanism of autolytic self-ingestion to decrease the thickness of the cell walls of *Nannochloropsis* microalgae (*Nannochloropsis* sp. and *Nannochloropsis gaditana*). The treatment consisted of a thermally coupled dark-anoxia incubation, which lead to the anaerobic metabolism being activated and the consequent consumption of sugar reserves. This resulted in the reduction of the polysaccharides comprised in the cellulose-based layer of the cell wall, whose thickness was then decreased to half. The process weakened the cells, which were then easier to rupture in subsequent treatments, as those previously mentioned for such purpose (in that specific case, high pressure homogenization).

## 3. Future Trends

The future of *Nannochloropsis* fatty acids extraction must, and certainly will, include efforts to comply with the principles defined in the 2030 Agenda for Sustainable Development [91]. In this regard, one issue that must be addressed is the economic impact of the extraction processes, which should be pondered when extraction conducted via a specific technology is studied. The energy requirements of a particular technology have a great impact on the overall cost of an extraction process, and the costs associated with the acquisition of the equipment for industrial production are also an important issue. This shows that the scalability of a process to an industrial setting entails several challenges of economic, environmental and sustainability natures. Oil separation and water removal are downstream processes whose improvement may significantly impact/reduce production costs. The possibility of reusing the solvents utilized in the extraction is another cost-reduction strategy, which will also decrease the environmental impact of the extraction process. Environmental concerns also guide the future of fatty acids extraction, as greener solvents as well as technologies that are able to increase the extraction yields of the conventionally utilized solvents are explored. The latter will allow to decrease the amount of solvent utilized in the extraction process, which will improve it in terms of costs and also regarding (by decreasing) its environmental impact. As previously mentioned, natural DES are considered promising solvents due to their sustainability traits. Modifications to DES and ILs may also increase their biodegradability and hence increase the opportunities to utilize such solvents. One other stage of the process that may be improved is the cell wall rupture, which is also an energy-costly process, and alternatives, such as auto-lysis, are being presented as the future path. Lastly, genetic engineering has been able to generate strains with increased fatty acid production which, consequently, will increase the yields of the extraction processes, thereby reducing the overall impact of obtaining similar amounts of the compounds.

## 4. Conclusions

Throughout the present review several technologies were described, which were (and still are) explored with the aim of reducing the negative environmental and health impacts of utilizing the conventional organic solvents in fatty acids extraction. The studies demonstrated that many technologies have the ability to improve fatty acids yields by either rupturing the cells and/or improving solvent penetration. Hence, the use of such technologies may allow the use of different “greener” solvents that have previously been dismissed due to lower extraction yields. Nonetheless, it was observed that some technologies are more prone to extract a specific type of fatty acids, and that in some cases a specific technology presented different impact on yields according to the *Nannochloropsis* species. One may therefore conclude that particular attention must be paid to the application of the technology, and that its impact on a specific fatty acid from a specific microalga must be assessed independently, in a case-by-case approach. It can also be inferred that combination of technologies may be the future path, since it allows for increased yields, as well as the recovery and separation of different compounds (such as distinct fatty acids), from a single sequential extraction procedure.

## Figures and Tables

**Table 1:** Recent studies utilizing microwave-assisted extraction to extract lipids and fatty acids from *Nannochloropsis* microalgae.

MW/IL/US	Species	Solvent	Operational Conditions			Yield						References
						Lipid	Fatty Acids					
			Used	Tested	Optimum		SFA	MUFA	PUFA	Omega-3	EPA	
MW + IL	*N*. *oceanica*	[TMam] [Cl]	1.65 g IL 2.45 GHz 700 W	60–90 °C; 1–25 min	90 °C; 25 min	―	―	―	―	―	37.92 mg g^−1^ FAME	[43]
MW + IL	*N*. *oceanica*	[TMam] [Cl]	2.45 GHz 700 W	0.5–2.5 g IL; 60–100 °C; 5–30 min	1.65 g IL; 88.2 °C; 24.7 min	19.6% (dw)	35.8 mg g^−1^ FAME	35.2 mg g^−1^ FAME	51.1 mg g^−1^ FAME	39.4 mg g^−1^ FAME	19.6 g g^−1^ biomass; 37.9 mg g^−1^ FAME	[28]
MW	*N*. *gaditana*	CHCl_3_:MeOH (2:1)	≤100 W	50–100 °C 5–25 min	100 °C; 5 min	17.7% (dw)	―	―	―	―	―	[29]
	*N*. *oceanica*				91 °C; 25 min	49% (dw)	―	―	―	―	―	
MW	*Nannochloropsis* sp.	Brine (NaCl) solution	2.45 GHz ≤800 W	60–100 °C 1–30 min	100 °C; 30 min	16.1% (dw)	~26% (TFA)	~23% (TFA)	44.1% (TFA); 25.2 mg g^−1^ (dw)	41.4% (TFA); 23.6 mg g^−1^ (dw)	31.5% (TFA); 17.9 mg g^−1^ (dw)	[40]
MW	*Nannochloropsis* sp.	Brine (NaCl) solution	2.45 GHz ≤ 800 W	1–35% (*w*/*v*) NaCl; 5–25% solid loading; 60–100 °C; 5–30 min	10% (*w*/*v*) NaCl; 5% solid loading; 100 °C; 30 min	16.1% (dw)	~27% (TFA)	~24% (TFA)	44.5% (TFA)	43% (TFA)	43% (TFA)	[44]
MW + IL	*Nannochloropsis* sp.	MeOH:[EMIM] [MeSO_4_]	2.45 GHz 700 W	4–12 (Alga:MeOH ratio); 0.5–1 (MeOH:IL ratio); 5–25 min	1:4 (Alga:MeOH ratio); 1:0.5 (MeOH:IL ratio); 25 min	42.2% (dw)	10.2% (ww)	18.7% (ww)	9.3% (ww)	―	―	[42]
MW	*N*. *oculata*	CHCl_3_:MeOH (2:1)	300 W	40–80 °C; 1–10 min	300 W; 80 °C; 1 min	33.6%	30.9% (TFA)	32.1% (TFA)	36.6% (TFA)	29.8% (TFA)	29.8% (TFA)	[45]
MW + US	*Nannochloropsis* sp.	MeOH	―	2:1–2:3 (Alga:MeOH) ratio; 100–140 W (US and MW); 3–7 min	2:3 (Alga:MeOH) ratio; 140 W; 7 min	FAME–48.2%	―	―	―	―	―	[41]

MW—microwaves; IL—ionic liquid; US—ultrasounds; SFA—saturated fatty acids; MUFA—monounsaturated fatty acids; PUFA—polyunsaturated fatty acids; EPA—eicosapentaenoic acid; TFA—total fatty acids; FAME—fatty acid methyl esters; [TMam] [Cl]—tetramethyl ammonium chloride; [EMIM] [MeSO_4_]—1-ethyl-3-methylimmidazolium methyl sulphate; CHCl_3_—chloroform; MeOH—methanol; dw—dry weight; ww—wet weight.

**Table 3:** Examples of supercritical fluid extraction of lipids and fatty acids from *Nannochloropsis*.

SF/PL/DEG	Species	Co-Solvent	Operational Conditions			Yield						References
						Lipid	Fatty Acids					
			Used	Tested	Optimum		SFA	MUFA	PUFA	Omega-3	EPA	
SF	*N*. *oculata*	*n*-hexane	200 mL min^−1^ (fr)	150–550 bar; 35–75 °C; 0–300 min; 0–3% co-solvent	550 bar; 75 °C; 150 min; 3% co-solvent	0.262 g g^−1^ (dw)	34.4% (TFA)	52.5% (TFA)	13.7% (TFA)	―	7.4% (TFA)	[30]
SF + PL	*Nannochloropsis* sp.	Ethanol	35 MPa; 50 °C; 10% co-solvent; 0.15 kg h^−1^ (fr)	―	―	―	―	―	―	―	61.9%	[12]
SF	*N*. *oculata*	Ethanol	―	0–80% water content; 100–200 bar; 100–150 °C; 80–160 min	40% water content; 150 °C; 150 bar; 120 min	0.253 g g^−1^ (dw)	59% (TFA)	35.6% (TFA)	5% (TFA)	―	―	[54]
SF	*N*. *maritima*	Ethanol	80 min; 0.02 kg h^−1^ (fr)	100–300 bar; 40–60 °C	300 bar; 40 °C	―	―	―	―	―	8.5% (total lipids)	[55]
SF + DEG	*Nannochloropsis* sp.	―	100 min	100–550 bar; 50–75 °C; 7.24–14.48 g min^−1^ (fr)	550 bar; 75 °C; 14.48 g min^−1^ (fr)–EPA 400 bar; 50 °C; 14.48 g min^−1^ (fr)–DHA	18.39 mg g^−1^ (dw)	4.74 mg g^−1^ (dw)	5.89 mg g^−1^ (dw)	6.92 mg g^−1^ (dw)	―	5.69 mg g^−1^ (dw)	[53]
SF + DEG	*N*. *gaditana*	―	100 min; 50 or 65 °C	250–550 bar; 7.24–14.48 g min^−1^ (fr)	250 bar; 65 °C; 7.24 g min^−1^ (fr)	34.61 mg g^−1^ (dw)	―	―	―	―	11.50 mg g^−1^ (dw)	[56]
SF	*N*. *oculata*	Ethanol	50 °C; 25 g min^−1^ (fr)	250–750 bar; 0–240 min	450 bar; 240 min	20%	―	―	―	―	―	[57]

SF—supercritical fluid; PL—pressurized liquid; DEG—diatomaceous earth grinding; SFA—saturated fatty acids; MUFA—monounsaturated fatty acids; PUFA—polyunsaturated fatty acids; EPA—eicosapentaenoic acid; DHA—docosahexaenoic acid; TFA—total fatty acids; fr—flow rate; dw—dry weight.

**Table 4:** Studies concerning *Nannochloropsis* extractions of lipids and fatty acids via pressurized liquid extraction.

Species	Co-Solvent	Operational Conditions			Yield						References
					Lipid	Fatty Acids					
		Used	Tested	Optimum		SFA	MUFA	PUFA	Omega-3	EPA	
*N*. *salina*	Ethanol	20 bar; 120 min	90–150 °C; 25–75% (water ethanol); 1–4 mL min^−1^ (fr)	90 °C; 75% (water ethanol); 4 mL min^−1^ (fr)	33.9% (dw)	44.26% (TFA)	41.35% (TFA)	14.39% (TFA)	―	―	[31]
*N*. *salina*	―	20 bar; 120 min	150–200 °C; 1–4 mL min^−1^ (fr); 1–4 g_sample_	175 °C; 4 mL min^−1^ (fr); 1 g_sample_	19.52% (dw)	35.48% (TFA)	53.11% (TFA)	11.41% (TFA)	―	―	[58]
*N*. *gaditana*	―	―	156.1–273.9 °C; 6.6–23.4 min; 33–117 g_sample_ L^−1^	236.54 °C; 13.95 min; 60.5 g_sample_ L^−1^	13.4% (dw)	―	―	―	―	15.04% (FAME)	[25]
*N*. *gaditana*	―	10 min	hexane or ethanol	hexane; 120 °C	17.6% (dw)	13.4% (TFA)	20.0% (TFA)	66.5% (TFA)	55.9% (FAME)	53% (FAME)	[11]
*N*. *gaditana*	―	120 °C; 15 min	Solvents	2-MeTHF:ethanol (1:3)	46.1% (dw)	20.17% (TFA)	27.86% (TFA)	51.94% (TFA)	39.73% (TFA)	38.54%	[23]

SFA—saturated fatty acids; MUFA—monounsaturated fatty acids; PUFA—polyunsaturated fatty acids; EPA—eicosapentaenoic acid; TFA—total fatty acids; FAME—fatty acid methyl esters; 2-MeTHF—2-Methyltetrahydrofuran; fr—flow rate; dw—dry weight.

**Table 5:** Enzyme-assisted extraction applied to *Nannochloropsis* to obtain lipids and fatty acids.

Enzymes	Species	Solvent (Post-Enzymes)	Operational Conditions			Yield						References
						Lipid	Fatty Acids					
			Used	Tested	Optimum		SFA	MUFA	PUFA	Omega-3	EPA	
Laccase and cellulase	*N. oceanica*	EtOH	―	Laccase:cellulase (4:1, 2:1, 1:1,1:2, 1:4); pH (4.2–5.8); T (40–60 °C); time (1.5–24 h)	1:2.5 (laccase:cellulase); pH 5; 45 °C; 6 h	26.9%	―	―	―	―	20.7 g 100 g^−1^	[20]
Cellulase, hemicellulase, papain and pectinase	*N*. *oculata*	―	200 U enzyme; pH 5.5; 45 °C; 12 h	Combinations	Mixture of all four	221.4 mg g^−1^ (dw)	―	―	―	―	―	[64]
Cellulase, hemicellulase, papain and pectinase	*Nannochloropsis* sp.	*tert*-butanol	200 U enzyme; pH 5.0; 50 °C; 4 h	Cellulase; hemicellulase; papain; pectinase	cellulase	88.70% (ww)	53% (TFA)	25.69% (TFA)	19.91% (TFA)	13.98% (TFA)	13.98% (TFA)	[35]
Cellulase and mannase	*Nannochloropsis* sp.	n-hexane:2-propanol (3:2)	13.8 mg g^−1^ (cellulase), 1.5 mg g^−1^ (mannase); pH 4.4; 53 °C; 24 h	Cellulase; mannase; cellulase/mannase	cellulase/mannase	73% (total lipids)	―	―	―	―	―	[60]

SFA—saturated fatty acids; MUFA—monounsaturated fatty acids; PUFA—polyunsaturated fatty acids; EPA—eicosapentaenoic acid; TFA—total fatty acids; EtOH—ethanol; ww—wet weight; dw—dry weight.

**Table 6:** *Nannochloropsis* lipids extracted using ionic liquids or deep eutectic solvents.

Species	IL or DES	Parameters		Lipid Yield	References
		Tested	Optimum		
*Nannochloropsis* sp.	Tetramethylguanidine:menthol	1:1–5:1 (HBA:HBD molar ratio); 50–90 °C; 30–110 min; 5–40% (ammonium sulfate); 20:1–40:1 (liquid:solid ratio)	3:1 (HBA:HBD molar ratio); 80 °C; 90 min; 20% (ammonium sulfate); 35:1 (liquid:solid ratio)	127 mg g^−1^ (dw)	[71]
*N*. *oceanica*	[EMIM]Cl	0.2–3 g IL	2 g IL	13.9%	[72]
*N*. *oculata*	Lactam- or ammonium carboxylate-based	ILs	butyrolactam hexanoate	13.5% (dw)	[69]
*Nannochloropsis* sp.	Cholinium amino acid-based	ILs	cholinium arginate	98.6% (total lipids)	[7]
*N*. *gaditana*	[EMIM] [MeSO_4_]	65–95 °C; 5–25 min; 1:4–1:12 (wet alga:MeOH); 1:0.5–1:1 (MeOH:IL)	14 min; 1:4 (wet alga:MeOH); 1:0.5 (MeOH:IL)	41.2% (dw)	[42]

IL—ionic liquid; DES—deep eutetic solvent; [EMIM] [MeSO_4_]—1-ethyl-3-methylimmidazolium methyl sulfate; [EMIM]Cl—1-ethyl-3-methyl imidazolium chloride; HBA—hydrogen-bond acceptor; HBD—hydrogen-bond donor; MeOH—methanol; dw—dry weight.

## Data Availability

The data used to support the findings of this study are available from the corresponding author upon request.
